# Investigation of Psychological Stress and Sleep Quality of Emergency Medical Technicians in Taiwan Fire Department during the COVID-19 Pandemic

**DOI:** 10.3390/ijerph20010137

**Published:** 2022-12-22

**Authors:** Chiao-Yin Cheng, Jen-Tang Sun, Hung-Pin Chang, Yen-Lin Chen, Dee Pei, Yao-Jen Liang

**Affiliations:** 1 Department of Emergency Medicine, Far Eastern Memorial Hospital, New Taipei 220, Taiwan; 2 Graduate Institute of Applied Science and Engineering, Fu Jen Catholic University, New Taipei 242, Taiwan; 3 Department of Nursing, Cardinal Tien Junior College of Healthcare and Management, Yilan 266, Taiwan; 4 Department of Pathology, Tri-Service General Hospital, Medical Defense Medical Center, Taipei 114, Taiwan; 5 Division of Endocrinology and Metabolism, Department of Internal Medicine, Fu Jen Catholic University Hospital, School of Medicine, College of Medicine, Fu Jen Catholic University, New Taipei 242, Taiwan

**Keywords:** EMS, DASS-21, PSQI, firefighter, COVID-19

## Abstract

When the coronavirus disease 2019 (COVID-19) began to ravage the world in 2019, the World Health Organization became concerned. The epidemic has a high mortality and contagion rate, with severe health and psychological impacts on frontline emergency medical service system practitioners. There are many hospital staff surveys, but few have covered the stress among emergency medical technicians. DASS-21, PSQI, and AUDIT questionnaires were used to evaluate the sources of psychological stress factors of firefighters in Taiwan. Multiple logistic regression was used to analyze the questionnaire content. We conducted questionnaire surveys from May 2022 to July 2022. Our sample comprised 688 participants. The odds ratios of increased depression, anxiety, and stress levels due to reduced family or peer understanding and support were 2.72 (95% CI: 1.50–4.92), *p* = 0.001; 2.03 (95% CI: 1.11–3.68), *p* = 0.021; and 3.27 (95% CI: 1.83–5.86), *p* < 0.001, respectively. The odds ratios of poor sleep quality due to depression, anxiety, and increased stress levels were 5.04 (3.18–7.99), *p* < 0.001; 2.44 (95% CI: 1.57–3.81), *p* < 0.001; and 4.34 (95% CI: 2.76–6.82), *p*-value < 0.001, respectively. During the COVID-19 pandemic, poor sleep quality and a lack of understanding and support from the Taiwan firefighting agency staff, family, or peers resulted in increased depression, anxiety, and stress levels.

## 1. Introduction

Coronavirus disease 2019 (COVID-19), also known as severe respiratory disease coronavirus 2 (SARS-CoV-2), began to ravage the world in 2019; the World Health Organization (WHO) declared the epidemic a public health emergency. The pandemic started in Wuhan, China, in December 2019 [1] and has severely damaged global economic, social, and health development [2,3]. The pandemic has seen high mortality and contagion rates, with health and psychological impacts on frontline emergency medical service system practitioners [4,5]. During the 2002 SARS outbreak, it was reported that the pandemic would cause sequelae of psychological stress among medical staff [6,7]. Similar results have been reported for the Ebola virus pandemic [8,9].

During the epidemic period in Taiwan, the general control measures of the second-level alert have been as follows: 1. Avoid entering and leaving crowded places and places with high risk of infection transmission. People must wear masks in accordance with regulations, temperature measurement, disinfection, crowd control, total volume control, moving line planning and other measures. 3. In principle, gatherings of more than 500 people outdoors and more than 100 people indoors are prohibited. 4. Eating and drinking are prohibited in public transportation, such as railways and passenger transportation. At that time, all people suspected or confirmed of being infected with COVID-19 were evacuated by emergency medical technicians affiliated to the fire department, and these personnel were equipped with a full set of waterproof isolation, N95-level masks, hair caps and shoe covers, and ordered to undergo relevant disinfection procedures.

In a 2020 Asian multicenter study, assessed using the Depression Anxiety Stress Scales-21 (DASS-21) questionnaire, the psychological stress of healthcare workers during the pandemic was significantly associated with the outbreak [10]. Another 2021 analysis of 609 medical and non-medical professionals in Europe using the DASS-21 questionnaire showed that a subset of people with depression, anxiety, and stress scores met the criteria for professional intervention [11].

Sufficient sleep can regulate the body’s blood immune cells and hormones. In addition, the correlation between sleep and mental health is also well documented [1,12,13]. In 2020 and 2021, many reports indicated that, through the analysis of sleep quality using the Pittsburgh Sleep Quality Index (PSQI) questionnaire, approximately 43.9% to 71.2% of doctors had sleep quality problems [14,15,16]. This issue was also noted in nursing staff; 45.7%~64.8% of frontline nurses in Turkey suffer from sleep problems [17,18,19]. Although psychological stress and sleep problems were closely related during the pandemic, other factors can still exacerbate both issues. These include being an older adult over 65 years of age at home, disease transmission to family members, death of patients infected with COVID-19, social support, stigma, and exposure to violence [20,21,22,23,24].

Alcoholism is a similar cause for concern. A survey of 189 first responders in the United States from June to August 2020 found that first responders exposed to COVID-19 reported higher levels of alcohol use [25]. Seven percent of UK intensive care staff were found to have alcohol-related problems [26]. Another report in the UK found that 10.5% of the 4378 UK healthcare and support staff had alcohol problems [27].

Taiwan’s pre-hospital emergency medical services are mainly composed of emergency medical technicians (EMTs) who belong to the National Fire Agency (NFA) and are life-long civil servants after passing the national examination. Their rotation system may vary slightly due to different regions or shifts with colleagues, but in principle, they work for 48 consecutive hours and rest for 24 h. Their job content is firefighting, emergency rescue and emergency medical services.

In addition, based on the above introduction, we take factors that may affect psychological stress as our variables, such as whether there are elderly people in the family, fear of infecting family members due to the nature of work, increase in the number of confirmed cases and deaths, stigma, subsequent analysis with violent incidents, sleep and alcohol problems, etc., to confirm whether these factors actually affect the individual’s psychological stress. Taiwan’s emergency medical service system practitioners include prehospital emergency medical technicians, doctors, and nurses. Most existing studies on psychological stress and sleep discuss doctors and nurses. However, EMTs involved before hospitalization were also exposed to the stressors of the COVID-19 pandemic, and their issues must also be addressed. There have been very few reports on this topic. We will use the DASS-21, PSQI, alcohol, and other related questions to explore the psychological stress, sleep quality, and alcoholism of EMTs during the pandemic. We provide the relevant results to the fire department for reference and formulate relevant pressure relief measures.

## 2. Materials and Methods

### 2.1. Questionnaire Background

**Figure 1:**
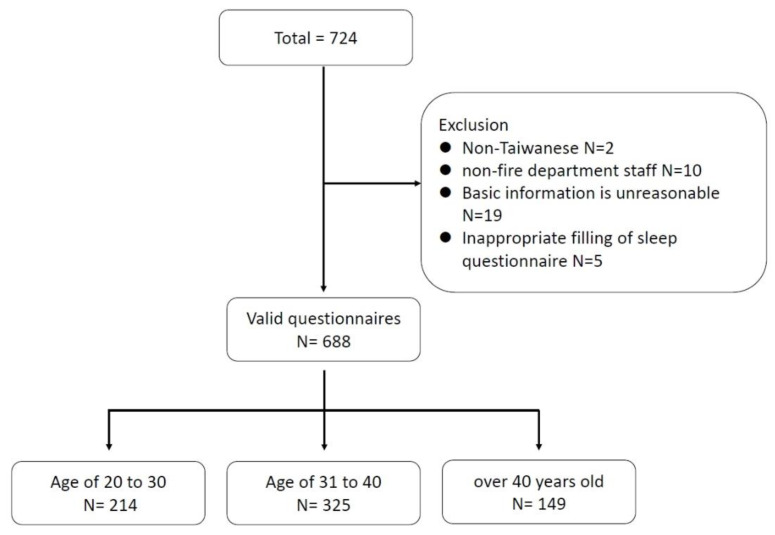
Diagram of the study participants’ selection process.

This questionnaire survey evaluated firefighting personnel working for the NFA from May to July 2022. This was a period when the Taiwanese government announced the COVID-19 pandemic level three alerts. Regions are divided into northern, eastern, central, and southern according to the number of firefighting establishments in each county and city. The content and methods of the questionnaire were approved by the institutional review board of the Far Eastern Memorial Hospital (number 111045-E). The paper and online questionnaires were collected simultaneously, and voluntary participants were recruited and filled in by local medical advisors. The inclusion criteria are that all EMTs belonging to NFA can fill out this questionnaire. Before filling out the questionnaire, there is a description of this study, and only if you agree to the content of the description can you fill it out; otherwise, you can refuse. We recruited a total of 724 firefighters, and 36 were excluded. The exclusion criteria were unreasonable basic information, non-firefighters, non-Taiwanese, and lack of sleep questionnaires. In total, 688 valid questionnaires were used in the first part of the analysis. In addition, age groups were subdivided into 20–30 years old, 31–40 years old, and at least 41 years old (Figure 1).

### 2.2. Questionnaire Design

The research questionnaire was written in Chinese and consisted of demographic characteristics, a family status survey, medical history, work type and hours, personal experience, DASS-21, PSQI, and The World Health Organization’s Alcohol Use Disorders Identification Test (AUDIT). The Chinese version of the above questionnaire was verified in previous studies. The relevance and clarity of the questionnaire content were verified by a psychiatrist, three emergency physicians, and two paramedics. The questionnaire was divided into three parts: basic information, DASS-21, PSQI, and AUDIT. DASS-21, PSQI and AUDIT Chinese questionnaires were verified in previous research, and the basic data are designed by us. The Cronbach’s alpha values of basic information, DASS-21, PSQI and AUDIT are 0.71, 0.96, 0.86 and 0.84, respectively.

### 2.3. DASS-21

The DASS-21 is an emotional scale for evaluating depression, anxiety, and stress. It uses 21 questions and sums the scores to understand an individual’s recent state of the three emotions. Depression was rated as mild (10–13), moderate (14–20), severe (21–27), or very severe (≥28). Anxiety was rated as mild (8–9), moderate (10–14), severe (15–19), or very severe (≥20). Stress was rated as mild (15–18), moderate (19–25), severe (26–33), or very severe (≥34). Generally, a moderate level or above can be a sufficient reason to seek specialist interventions [28].

### 2.4. Variables

We will include region, age, gender, education level, marital status, education level, past medical history, psychiatric history, working years, whether there are any family members who are younger than 18 years old or older than 65 years old, weekly working hours, whether the work unit refers to the transport and treatment of suspected or confirmed patients, the number of suspected or confirmed patients who have been transported when filling out the questionnaire, whether they have experienced SARS, whether they are worried about infecting family members because of work, whether they want to temporarily live apart from their families because of work, whether violent or stigmatized incidents were experienced, mediocre media reports, the continuous increase in the number of confirmed cases and deaths, the lack of personal protective equipment, the lack of vaccines and therapeutic drugs, and the decrease in support and understanding of family members and peers as are our variables.

### 2.5. PSQI

The PSQI questionnaire is composed of 19 items to generate seven scores for the following factors: sleep quality, sleep latency, sleep duration, habitual sleep efficiency, sleep disturbance, sleep medication use, and daytime dysfunction. All scores were summed to obtain a total score. The higher the score, the worse the sleep quality. Scores over five points are defined as poor sleep quality [29].

### 2.6. AUDIT

The AUDIT is a 10-question questionnaire with a total score ranging from 0 to 40. It investigates the frequency, quantity, and dependence on drinking. The higher the score, the greater the degree of alcohol dependence. A score of eight or higher indicates drinking problems [30].

### 2.7. Statistics

The data were analyzed using IBM SPSS Statistics 26 (IBM Corp., Armonk, NY, USA). The Kolmogorov–Smirnov test was used to verify the normal distribution. None of our variables were normally distributed; therefore, the Mann–Whitney U test was used. We first performed a univariate logistic regression analysis on the analyzed variables and then performed a multivariate logistic regression analysis on variables with significant differences. We forced the addition of basic information, such as age, sex, and medical history, among others. A *p*-value < 0.05 indicates a significant difference.

## 3. Results

The average age of surveyed participants was 34.9 years, with 652 men and 36 women. We used 26 basic information items as variables. Moreover, we noted the ratio of variables with a depression score ≥14, anxiety score ≥10, and stress score ≥19. Most participants were located in the north, east, and island regions; 380 total. Of these, 152 (39.0%) had depression scores ≥14, 170 (43.6%) had anxiety scores ≥10, and 120 (30.8%) had stress scores ≥19. Of the 217 participants in the central region, 87 (40.1%) had depression scores ≥14, 93 (42.9%) had anxiety scores >10, and 57 (26.3%) had stress scores ≥19. Of the 81 participants in the southern region, 45 (55.6%) had depression scores ≥14, 48 (59.3%) had anxiety scores ≥10, and 35 (43.2%) had stress scores ≥19. A total of 81 participants felt that their family members or peers were concerned, and their body weight decreased during the pandemic; 58 (71.6%) had a depression score ≥14, 59 (72.8%) had an anxiety score ≥10, and 55 (67.9%) had a stress score ≥19. A total of 141 participants had poor sleep quality; 104 (73.8%) had a depression score ≥14, 96 (68.1%) had an anxiety score ≥10, and 85 (60.3%) had a stress score ≥ 19 (Table S1).

Depression scores differed significantly by region, age, sex, work experience, and living with family members <18 years. Anxiety scores varied significantly by region, age, sex, marital status, work experience, and living with family members <18 years. Stress scores varied significantly by region, age, sex, work experience, and living with family members <18 or >65 (Table 1). Furthermore, we investigated other variables. Among the variables that affect depression, anxiety, and stress scores are hours worked per week, number of COVID-19 patients transported, concern about spreading the virus to your family, finding alternate accommodation to live apart from your family due to work, increase in deaths or diagnoses, exposure to violence or stigma, personal protective equipment, lack of treatment, lack of medication, less concern or consideration from peers and family members, problematic alcohol use, and sleep quality. Frequent media coverage of COVID-19-related events and lack of vaccines affected anxiety and stress scores but not depression scores (Table 2).

We discuss the variables that affect depression, anxiety, and stress scores in Table 1 and Table 2 and perform univariate logistic regression analysis. Univariate logistic regression analysis was performed for variables with a *p*-value < 0.05. This was followed by multivariate logistic regression analysis with the mandatory addition of basic personal information.

The odds ratio for depression score ≥14 was 2.63 times higher in southern regions than in northern, eastern, and island regions (95% CI 1.47–4.72), *p* = 0.001. The odds ratios for work experience of 6–10 years and 10–15 years were 2.16 (95% CI: 1.26–3.71), *p* = 0.005, and 3.01 (95% CI: 1.53–5.93), *p* = 0.005, respectively; there was no difference between groups >15 years. The odds ratio for those working 72–96 h per week is 1.76 (95% CI: 1.00–3.07), *p* = 0.049, higher than those working >96 h and <72 h. Those who perceived less understanding and support from their family members or peers were 2.72 (95% CI: 1.50–4.92) times more likely than those who perceived no change in support, *p* = 0.001. The odds ratio for those with poor sleep quality was 5.04 (3.18–7.99), *p* < 0.001. (Table 3)

The odds ratio for anxiety scores ≥10 was 2.77 (95% CI: 1.54–4.99) times higher in the southern region than in the northern, eastern, and island regions, *p* = 0.001. Men had lower odds ratios than women, with an odds ratio of 0.28 (95% CI: 0.12–0.65), *p* = 0.003. Those who worked >96 h per week had higher odds than those who worked fewer than 72 h, with an odds ratio of 1.85 (95% CI: 1.03–3.33), *p* = 0.041. Those who perceived less understanding and support from their family members or peers were 2.03 (95% CI: 1.11–3.68) times more likely than those who perceived no change in support, *p* = 0.021. The odds ratio for those with poor sleep quality was 2.44 (95% CI: 1.57–3.81), *p* < 0.001. (Table 4)

The odds ratio for a stress score ≥19 points was 2.78 (95% CI: 1.50–5.14) times higher in the southern region than in the northern, eastern, and island regions, *p*-value = 0.001. During the pandemic, the odds ratio of transporting 21–40 suspected or confirmed COVID-19 patients compared with ≤20 patients was 1.60 (95% CI: 1.02–2.51), *p*-value = 0.04. Those who perceived less understanding and support from their family members or peers were 3.27 (95% CI: 1.83–5.86) times more likely than those who perceived no change in support, *p*-value < 0.001. The odds ratio for those with poor sleep quality was 4.34 (95% CI: 2.76–6.82), *p*-value < 0.001 (Table 5).

**Figure 2:**
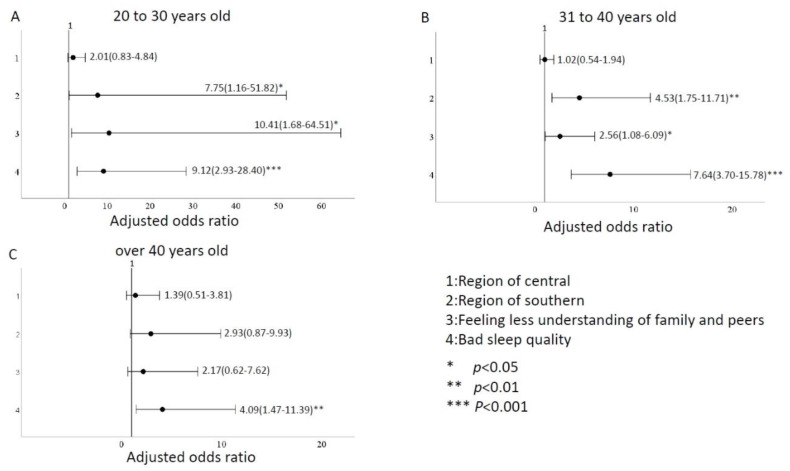
Comparison of odds ratio factors for depression levels at different ages. (**A**) between 20–30 years old (**B**) between 31–40 years old (**C**) over 40 years old.

**Figure 3:**
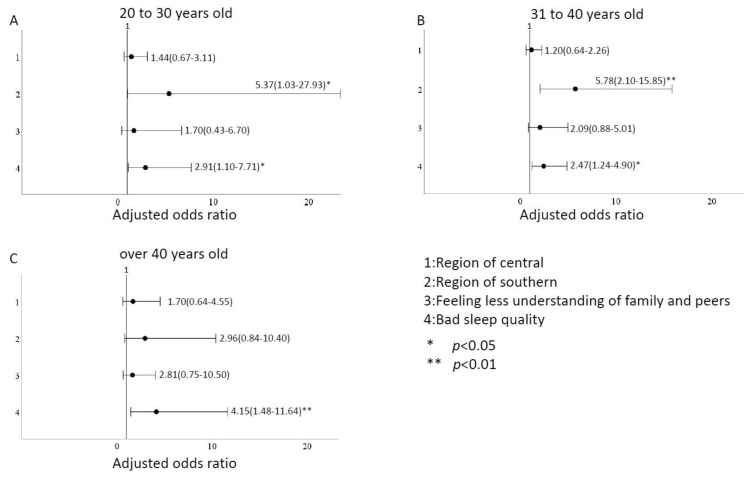
Comparison of odds ratio factors for anxiety levels in different ages. (**A**) between 20–30 years old (**B**) between 31–40 years old (**C**) over 40 years old.

**Figure 4:**
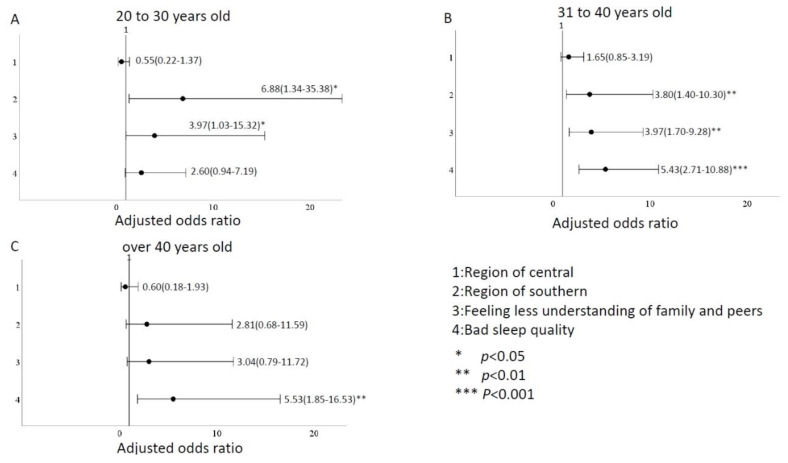
Comparison of odds ratio factors for stress levels in different ages. (**A**) between 20–30 years old (**B**) between 31–40 years old (**C**) over 40 years old.

We divided ages into subgroups of 20–30 years old, 31–40 years old, and >40 years old for analysis. In addition, we described our subgroups with a total of 214 people aged 20–30, with an average age of 26.9 years, 126 (58.9%) in the northern region, 75 (35.0%) in the central region and 13 (6.1%) in the southern region. 16 (7.5%) felt that family members would have less understanding and support from their peers, and 29 (13.6%) had poor sleep quality. A total of 325 people aged 30–40, with an average age of 35.5 years old, 198 (60.9%) in the northern region, 98 (30.2%) in the central region and 29 (8.9%) in the southern region. 45 (13.8%) felt that family members would have less understanding and support from their peers, and 73 (22.5%) had poor sleep quality. A total of 149 people were over 40 years old, with an average age of 45.2 years, 66 (44.3%) in the northern region, 44 (29.5%) in the central region and 39 (26.2%) in the southern region. 20 (13.4%) felt that family members would have less understanding and support from their peers, and 39 (26.2%) had poor sleep quality. Using the same variables as in Table 3, multivariate logistic regression was used to analyze the influence of different age groups on depression. We found that firefighters in their 20s and 30s, working in the southern region, with less understanding or support from peers and family members and poor sleep quality had moderate to higher depression levels than other age groups; the odds ratios were 7.75 (95% CI:1.16–51.82), 10.41 (95% CI:1.68–64.51), and 9.12 (95% CI:2.93–28.40), respectively (Figure 2). Firefighters in their 20s and 40s in the southern region were more prone to anxiety than those in the north, east, and island regions. The odds ratios were 5.37 (1.03–27.93) for the 20–30-year-olds and 5.78 (2.10–15.85) for the 30–40-year-olds. Poor sleep quality was also one of the variables that increased the odds ratio for anxiety levels, especially for those over 40, with an odds ratio of 4.15 (1.48–11.64), as shown in Figure 3. Firefighters aged 20–40 in the southern region are more prone to stress than those in the north, east, and island regions, with odds ratios ranging from 5.37 to 5.78. Firefighters in their 20s and 40s experienced heightened stress due to less understanding and support from their families and peers. Age groups 20–30, 31–40, and over 40 were more stressed due to poor sleep quality; the odds ratios for the medium and above were 2.91 (1.10–7.71), 2.47 (1.24–4.90), and 4.15 (1.48–11.64), respectively (Figure 4).

## 4. Discussion

From May to July 2022, during COVID-19 alert level 3 in Taiwan, firefighters working in the southern region had higher levels of depression, anxiety, and stress than those working in the north, east, and island regions. In addition, the number of hours worked per week, less understanding or support from family and peers, and poor sleep quality increased the odds of higher levels of depression, anxiety, and stress. We believe that the odds ratio of firefighters in the southern region was higher than in other places because they had not yet experienced a severe wave of COVID-19. The northern regions had already experienced a wave of COVID-19 in 2021. When the pandemic recurs in 2022, the response mechanisms in the north will be prepared to handle the wave of infections.

According to subgroup analysis, firefighters aged 20–30 were more likely to have higher depression than other age groups due to work area, family and peer factors, and sleep quality. Firefighters in their 20s and 40s working in the southern region had a higher odds ratio for anxiety levels than firefighters in the north, east, and island regions. The odds ratio of firefighters over 40 years experiencing increased anxiety due to poor sleep quality was 1.42 to 1.68 times that of other age groups. Southern region firefighters aged 20–40 were more stressed than those in the north, east, and island regions. They also experienced heightened stress because they perceived less understanding and support from their families and peers. Firefighters over 30 experienced heightened stress due to poor sleep.

Since the outbreak of COVID-19, there have been several investigations into psychological stress among emergency medical service personnel. However, few investigations have been conducted on prehospital professionals. In 2021, Möckel et al. proposed a relationship between pain, pain medication use, and psychological stress of prehospital emergency medical service personnel [31]. In 2022, Mohammadreza Sabbaghi et al. surveyed 544 prehospital medical staff in eastern Iran. The results showed that, during the pandemic, medical staff experienced significant psychological pressure before arriving at the hospital. They determined that prehospital staff should be given more time for family communication and rest [32]. In the same year, Raúl Soto-Cámara et al. surveyed professionals in the emergency medical system in Spain. They indicated that emergency medical technicians were more psychologically stressed than other professionals [3]. In an Iranian study, ethnic groups similar to those in Taiwan suggested that psychological stress was significantly associated with working hours per week and living away from family; sleep quality was not mentioned as a factor.

According to a 2021 study in China and India, 3228 samples collected in Wuhan and 13,641 collected in India indicated that people who perceived less family or social support had higher scores for depression, anxiety, and stress [33,34]. These results are similar to those of our study. Poor sleep quality was a risk factor in the present study. Chen’s survey of 248 frontline nurses who tested nucleic acids in China in 2021 showed that poor sleep quality was significantly related to depression, anxiety, and stress [35]. An Italian article in 2020 showed that 123 of the 432 samples were from medical staff, and the remaining 309 were from non-medical staff. Using the PSQI to evaluate the sleep quality score of medical staff resulted in 6.8 points, and that of non-medical staff was 5.1 points. The sleep quality of the medical staff was significantly worse than that of the non-medical staff. Poor sleep quality can lead to higher depression, anxiety, and stress scores [36].

The firefighting profession is one of the most dangerous and stressful occupations. However, existing literature has little empirical evidence examining the effects of stress on firefighters and emergency medical services (EMS) personnel. Recent studies have shown that firefighters are at increased risk for adverse mental health conditions, such as trauma syndrome (and depression). Repeated exposure to traumatic and critical events puts firefighters/EMS personnel at higher risk of suicide [37,38]. Moreover, firefighters have shown comparable or higher levels of suicidal ideation or attempted suicide compared to other high-risk service occupations. There are 46.8% [39,40]. Several studies have reported on suicide rates among veterans in the US. Veterans’ suicidal ideation was between 3.8% and 13.9%, attempted suicide was 4%-12.4%, and planned suicide was 5.3%. These rates are similar to the general population but still lower than among firefighters [41,42].

Frontline prevention personnel have been under considerable pressure during the COVID-19 pandemic. Our results indicate that poor sleep quality and a perceived decrease in understanding and support from family and peers contribute to increased depression, anxiety, and stress. We recommend that firefighters be given sufficient rest and timely care when unwell. Quality sleep conditions must be provided to reduce the impact of depression, anxiety, and stress. Moreover, firefighters must be provided with the necessary psychological assistance and support to reduce negative emotions.

This study was originally designed as a multi-country questionnaire, but it was not easy to collect online questionnaires, so in the end, only the data from Taiwan were analyzed. In addition, because there are few relevant literature studies on pre-hospital emergency medical technicians, we tried our best to search for references. Most of our variables are supported by previous studies, and a few are still not, but we used the Delphi method for expert evaluation to obtain relatively objective information, opinions, and insights, aiming to be objective and discriminative in the selection of variables. The original questionnaire design did not include whether the respondents were infected with COVID-19, a variable that may affect the severity of psychological stress. In addition, the number of samples in the northern region accounted for the majority in this study, and the southern region was slightly insufficient. However, we still tried our best to complete the analysis and provide valuable questionnaire survey data for Taiwan’s firefighting agencies.

## 5. Conclusions

During the COVID-19 pandemic, poor sleep quality and a lack of understanding and support from firefighting agency staff, family, or peers in Taiwan has led to increased depression, anxiety, and stress levels. We provide this study to the Taiwan fire department so that it can formulate strategies to promote the mental health of its personnel.

## Figures and Tables

**Table 1:** Average scores for depression, anxiety, and stress based on demographic information.

Variable		Depression		Anxiety		Stress	
		Mean (IQR)	*p*-Value	Mean (IQR)	*p*-Value	Mean (IQR)	*p*-Value
All		11.4 (12.0)		9.5 (12.0)		14.5 (14.0)	
Region	North, East, and Islands N = 390 (56.7%)	11.4 (14.5)	0.032 *	9.3 (12.0)	0.012 *	14.6 (14.0)	0.015 *
	Central N = 217 (31.5%)	10.7 (14.0)		8.8 (12.0)		13.4 (16.0)	
	Southern N = 81 (11.8%)	13.3(13.0)		12.0 (13.0)		17.0 (17.0)	
Age	20–30 years N = 214 (31.1%)	8.4 (14.0)	<0.001 ***	7.0 (10.0)	<0.001 ***	11.7 (14.0)	<0.001 ***
	31–40 years N = 325 (47.2%)	12.6 (14.0)		10.4 (12.0)		15.8 (14.0)	
	>40 years N = 149 (21.7%)	13.1 (12.0)		10.6 (12.0)		15.8 (15.0)	
Gender	Woman N = 36 (5.2%)	13.5 (8.0)	0.031 *	11.7 (10.0)	0.011 *	17.0 (8.0)	0.045 *
	Male N = 652 (94.8%)	11.3 (14.0)		9.3 (12.0)		14.4 (14.0)	
Marital status	Single N = 288 (41.9%)	10.6 (14.0)	0.067	8.6 (12.0)	0.038 *	13.5 (14.0)	0.067
	Divorce N = 20 (2.9%)	11.6 (15.0)		9.8 (11.0)		15.4 (18.5)	
	Married N = 380 (55.2%)	12.0 (14.0)		10.1 (12.0)		15.3 (14.0)	
Level of education	High school N = 21 (3.1%)	8.3 (13.0)	0.078	7.8 (11.0)	0.123	12.0 (15.0)	0.242
	College N = 270 (39.2%)	10.6 (14.0)		8.7 (6.0)		13.9 (14.0)	
	University N = 261 (37.9%)	11.9 (14.0)		10.1 (12.0)		15.1 (14.0)	
	Masters and Doctorate N = 136 (19.8%)	12.5 (12.0)		9.9 (12.0)		15.0 (14.0)	
History of disease	No N = 674 (98.0%)	11.4 (12.0)	0.075	9.5 (12.0)	0.601	14.5 (14.0)	0.698
	Yes N = 14 (2.0%)	12.9 (18.0)		8.1 (9.5)		15.6 (19.5)	
Any history of psychiatric illness in the past	No N = 680 (98.8%)	11.4 (14.0)	0.551	9.4 (12.0)	0.133	14.5 (14.0)	0.220
	Yes N = 8 (1.2%)	13.0 (11.0)		14.5 (20.5)		18.3 (11.5)	
Work experience	≤5 years 216 (31.4%)	8.1 (12.0)	<0.001 ***	7.2 (10.0)	<0.001 ***	11.5 (14.0)	<0.001 ***
	6–10 years N = 155 (22.5%)	12.4 (14.0)		10.0 (12.0)		15.2 (14.0)	
	10–15 years N = 165 (24.0%)	13.9 (14.0)		11.2 (12.0)		17.0 (14.0)	
	≥15 years N = 152 (22.1%)	12.4 (14.0)		10.3 (14.0)		15.4 (12.0)	
Living with family members under the age of 18	No N = 384 (55.8%)	10.2 (14.0)	<0.001 ***	8.3 (10.0)	<0.001 ***	13.2 (16.0)	<0.001 ***
	Yes N = 304 (44.2%)	12.9 (14.0)		10.9 (12.0)		16.1 (16.0)	
Living with family members over the age of 65	No N = 502 (73.0%)	11.0 (14.0)	0.080	9.2 (12.0)	0.112	14.0 (14.0)	0.016 *
	Yes N = 186 (27.0%)	12.4 (14.0)		10.3 (12.0)		15.8 (14.0)	

* *p* < 0.05; *** *p* < 0.001.

**Table 2:** Average scores for depression, anxiety, and stress according to different work experiences and exposure events.

Variable		Depression		Anxiety		Stress	
		Mean ± SD	*p*-Value	Mean ± SD	*p*-Value	Mean ± SD	*p*-Value
Average working hours per week	≤72 h N = 95 (13.8%)	8.7(12.0)	0.013 *	7.7(6.0)	0.014 *	11.8(16.0)	0.005 **
	72~96 h N = 400 (58.1%)	11.6(12.0)		9.2(12.0)		14.5(12.0)	
	>96 h N = 193 (28.1%)	12.4 (14.0)		10.9(12.0)		15.8(16.0)	
The work unit is dedicated to transporting confirmed or suspected infected patients	No N = 509 (74.0%)	11.5 (14.0)	0.720	9.6(12.0)	0.866	14.4(15.0)	0.467
	Yes N = 179 (26.0%)	11.1 (12.0)		9.1(12.0)		14.9(12.0)	
Transport or interact with this many suspected or COVID-19-positive patients	≤20 N = 454 (66.0%)	10.4 (14.0)	<0.001 ***	8.8(12.0)	0.003 **	13.2(14.0)	<0.001 ***
	21~40 N = 158 (23.0%)	12.8 (14.0)		10.7(12.0)		16.4(12.0)	
	>40 N = 76 (11.0%)	14.5 (13.5)		11.0(8.0)		18.2(12.0)	
Did you contract the severe acute respiratory syndrome (SARS) in 2002?	No N = 571 (83.0%)	11.2 (14.0)	0.260	9.3(12.0)	0.496	14.5(14.0)	0.976
	Yes N = 117 (17.0%)	12.4 (14.0)		10.1(14.0)		14.8(16.0)	
Do you worry about spreading the disease to your family due to work during the COVID-19 pandemic?	No N = 85 (12.4%)	7.7 (14.0)	<0.001 ***	5.9 (10.0)	<0.001 ***	8.9 (9.0)	<0.001 ***
	Yes N = 603 (87.6%)	11.9 (14.0)		10.0 (10.0)		15.3 (14.0)	
Do you want to find alternate accommodation and temporarily live apart from your family because of your work during the COVID-19 pandemic?	No N = 388 (56.4%)	10.2 (14.0)	<0.001 ***	8.2 (8.0)	<0.001 ***	12.8 (14.0)	<0.001 ***
	Yes N = 300 (43.6%)	13.0 (14.0)		11.1 (12.0)		16.7 (16.0)	
Have you experienced violence because of work?	No N = 560 (81.4%)	10.2 (14.0)	<0.001 ***	8.4 (12.0)	<0.001 ***	13.3 (14.0)	<0.001 ***
	Yes N = 128 (18.6%)	16.6 (17.5)		13.9 (14.0)		19.8 (16.0)	
Have you suffered from stigmatization because of your work?	No N = 533 (77.5%)	9.9 (14.0)	<0.001 ***	8.2 (12.0)	<0.001 ***	12.8 (14.0)	<0.001 ***
	Yes N=155 (22.5%)	16.7 (16.0)		13.7 (12.0)		20.4 (14.0)	
Are you worried about the frequent reports of COVID-19 in the media?	No N = 342 (49.7%)	10.7 (14.0)	0.092	8.8 (12.0)	0.024 *	13.5 (16.0)	0.014 *
	Yes N = 346 (50.3%)	12.1 (12.0)		10.1 (10.5)		15.5 (14.0)	
Are you concerned about the increased number of COVID-19-positive patients or deaths?	No N = 246 (35.8%)	9.4 (14.0)	<0.001 ***	7.2 (10.0)	<0.001 ***	11.1 (12.5)	<0.001 ***
	Yes N = 442 (64.2%)	12.5 (14.0)		10.7 (12.0)		16.4 (16.0)	
Do you worry about the lack of personal protective equipment, which increases the risk of exposure when attending work?	No N = 270 (39.2%)	9.0 (14.0)	<0.001 ***	7.1 (10.0)	<0.001 ***	11.5 (12.5)	<0.001 ***
	Yes N = 418 (60.8%)	13.0 (14.0)		11.0 (12.0)		16.4 (14.0)	
Are you worried about the lack of a COVID-19 vaccine?	No N = 381 (55.4%)	10.7 (14.0)	0.057	8.8 (12.0)	0.018 *	13.5 (14.0)	0.002 **
	Yes N = 307 (44.6%)	12.3 (14.0)		10.3 (12.0)		15.8 (14.0)	
Are you worried about the lack of drugs to treat COVID-19?	No N = 288 (41.9%)	9.9 (14.0)	0.001 **	8.1 (10.0)	<0.001 ***	12.6 (13.5)	<0.001 ***
	Yes N = 400 (58.1%)	12.5 (14.0)		10.4 (12.0)		15.9 (14.0)	
Do you believe the understanding and support of your family members or peers have diminished due to the pandemic?	No N = 607 (88.2%)	10.4 (14.0)	<0.001 ***	8.5 (12.0)	<0.001 ***	13.5 (14.0)	<0.001 ***
	Yes N = 81 (11.8%)	18.7 (16.0)		16.4 (17.0)		22.4 (16.0)	
Drinking problem	No N = 511 (79.5%)	10.9 (14.0)	0.015 *	8.8 (12.0)	<0.001 ***	13.9 (14.0)	0.004 **
	Yes N = 177 (25.7%)	12.9 (14.0)		11.3 (12.0)		16.3 (15.0)	
Sleep quality	Good N = 547 (79.5%)	9.4 (12.0)	<0.001 ***	7.9 (10.0)	<0.001 ***	12.5 (14.0)	<0.001 ***
	Bad N = 141 (20.5%)	19.3 (14.0)		15.7 (14.0)		22.2 (16.0)	

* *p* < 0.05; ** *p* < 0.01; *** *p* < 0.001.

**Table 3:** Depression odds ratio.

Variable		Univariable		Multivariable	
		OR (95% CI)	*p*-Value	OR (95% CI)	*p*-Value
Region	North, East, and Islands	Reference		Reference	
	Central	1.05 (0.75–1.47)	0.787	1.36 (0.90–2.05)	0.142
	Southern	1.96 (1.21–3.17)	0.006 **	2.63 (1.47–4.72)	0.001 **
Age	20–30 years	Reference		Reference	
	31–40 years	2.33 (1.61–3.39)	<0.001 ***	1.56 (0.88–2.77)	0.128
	>40 years	2.73 (1.76–4.24)	<0.001 ***	1.96 (0.90–4.27)	0.092
Gender	Woman	Reference		Reference	
	Man	0.61 (0.31–1.20)	0.153	0.64 (0.29–1.42)	0.273
Marital status	Single	Reference		Reference	
	Divorce	1.16 (0.46–2.93)	0.751	0.35 (0.11–1.09)	0.070
	Married	1.43 (1.04–1.95)	0.027 *	0.67 (0.39–1.15)	0.148
Level of education	High school	Reference		Reference	
	College	1.16 (0.45–2.97)	0.76	0.88 (0.30–2.55)	0.806
	University	1.68 (0.66–4.29)	0.281	1.10 (0.38–3.21)	0.866
	Masters and Doctorate	1.53 (0.58–4.04)	0.388	0.70 (0.23–2.16)	0.535
History of disease	No	Reference		Reference	
	Yes	1.43 (0.50–4.13)	0.505	1.46 (0.42–5.10)	0.555
Any history of psychiatric illness in the past?	No	Reference		Reference	
	Yes	1.43 (0.35–5.76)	0.616	1.16 (0.24–5.67)	0.860
Work experience	≤5 years	Reference		Reference	
	6–10 years	2.14 (1.38–3.30)	0.001 **	2.16 (1.26–3.71)	0.005 **
	10–15 years	2.97 (1.94–4.55)	<0.001 ***	3.01 (1.53–5.93)	0.001 **
	≥15 years	2.21 (1.43–3.43)	<0.001 ***	1.76 (0.81–3.84)	0.154
Living with family members under the age of 18	No	Reference		Reference	
	Yes	1.57 (1.16–2.13)	0.004 **	0.97 (0.61–1.54)	0.891
Living with family members over the age of 65	No	Reference		Reference	
	Yes	1.45 (1.03–2.03)	0.034 *	1.29 (0.87–1.93)	0.211
Average working hours per week	≤72 h	Reference		Reference	
	72~96 h	1.70 (1.05–2.75)	0.030 *	1.76 (1.00–3.07)	0.049 *
	>96 h	1.75 (1.04–2.95)	0.035 *	1.66 (0.90–3.06)	0.103
Transport or interact with this many suspected or COVID-19-positive patients	≤20	Reference		Reference	
	21~40	1.45 (1.00–2.09)	0.047 *	1.39 (0.91–2.13)	0.133
	>40	2.08 (1.28–3.40)	0.003 **	1.57 (0.88–2.81)	0.129
Do you worry about spreading the disease to your family due to work during the COVID-19 pandemic?	No	Reference		Reference	
	Yes	1.70 (1.04–2.77)	0.034 *	1.35 (0.74–2.46)	0.325
Have you experienced violence because of work?	No	Reference		Reference	
	Yes	2.28 (1.55–3.37)	<0.001 ***	1.42 (0.87–2.33)	0.165
Have you suffered from stigmatization because of your work?	No	Reference		Reference	
	Yes	2.34 (1.62–3.36)	<0.001 ***	1.09 (0.67–1.76)	0.729
Do you worry about the lack of personal protective equipment, which increases the risk of exposure when attending work?	No	Reference		Reference	
	Yes	1.73 (1.26–2.38)	0.001 **	1.06 (0.70–1.57)	0.792
Are you worried about the lack of drugs to treat COVID-19?	No	Reference		Reference	
	Yes	1.38 (1.01–1.88)	0.043 *	0.96 (0.65–1.42)	0.832
Do you believe the understanding and support of your family members or peers have diminished due to the pandemic?	No	Reference		Reference	
	Yes	4.25 (2.55–7.08)	<0.001 ***	2.72 (1.50–4.92)	0.001 ***
Drinking problem	No	Reference		Reference	
	Yes	1.45 (1.03–2.04)	0.035 *	1.13 (0.76–1.69)	0.546
Sleep quality	Good	Reference		Reference	
	Bad	5.73 (3.78–8.68)	<0.001 ***	5.04 (3.18–7.99)	<0.001 ***

* *p* < 0.05; ** *p* < 0.01; *** *p* < 0.001.

**Table 4:** Anxiety odds ratio.

Variable		Univariable		Multivariable	
		OR (95% CI)	*p*-Value	OR (95% CI)	*p*-Value
Region	North, East, and Islands	Reference		Reference	
	Central	0.97 (0.69–1.36)	0.861	1.24 (0.84–1.84)	0.288
	Southern	1.88 (1.16–3.06)	0.011 *	2.77 (1.54–4.99)	0.001 **
Age	20–30 years	Reference		Reference	
	31–40 years	2.11 (1.48–3.03)	<0.001 ***	1.77 (1.01–3.10)	0.046
	>40 years	2.37 (1.54–3.65)	<0.001 ***	2.00 (0.93–4.28)	0.075
Gender	Woman	Reference		Reference	
	Man	0.34 (0.17–0.71)	0.004 **	0.28 (0.12–0.65)	0.003 ***
Marital status	Single	Reference		Reference	
	Divorce	1.87 (0.75–4.64)	0.180	1.09 (0.36–3.28)	0.884
	Married	1.46 (1.07–2.00)	0.016 *	0.79 (0.47–1.36)	0.398
Level of education	High school	Reference		Reference	
	College	1.67 (0.66–4.28)	0.282	1.46 (0.50–4.29)	0.492
	University	1.81 (0.71–4.63)	0.216	1.13 (0.38–3.33)	0.832
	Masters and Doctorate	1.44 (0.55–3.80)	0.458	0.62 (0.20–1.93)	0.408
History of disease	No	Reference		Reference	
	Yes	0.91 (0.31–2.64)	0.859	0.73 (0.21–2.55)	0.622
Any history of psychiatric illness in the past?	No	Reference		Reference	
	Yes	2.04 (0.48–8.59)	0.333	2.24 (0.45–11.06=9)	0.323
Work experience	≤5 years	Reference		Reference	
	6–10 years	1.91 (1.25–2.91)	0.003 **	1.50 (0.89–2.53)	0.131
	10–15 years	2.63 (1.73–3.99)	<0.001 ***	1.85 (0.96–3.58)	0.069
	≥15 years	2.03 (1.33–3.11)	0.001 **	1.28 (0.60–2.72)	0.534
Living with family members under the age of 18	No	Reference			
	Yes	1.67 (1.24–2.27)	0.001 **	1.05 (0.66–1.66)	0.835
Average working hours per week	≤72 h	Reference		Reference	
	72~96 h	1.51 (0.95–2.41)	0.08	1.66 (0.97–2.84)	0.066
	>96 h	1.70 (1.03–2.82)	0.039 *	1.85 (1.03–3.33)	0.041 *
Transport or interact with this many suspected or COVID-19-positive patients	≤20	Reference		Reference	
	21~40	1.42 (0.98–2.04)	0.061	1.35 (0.89–2.05)	0.154
	>40	1.95 (1.19–3.18)	0.008 ***	1.45 (0.81–2.60)	0.206
Do you worry about spreading the disease to your family due to work during the COVID-19 pandemic?	No	Reference		Reference	
	Yes	2.47 (1.49–4.08)	<0.001 ***	1.68 (0.90–3.14)	0.103
Do you want to find alternate accommodation and temporarily live apart from your family because of your work during the COVID-19 pandemic?	No	Reference		Reference	
	Yes	1.63 (1.20–2.21)	0.002 **	1.06 (0.74–1.52)	0.763
Have you experienced violence because of work?	No	Reference		Reference	
	Yes	2.69 (1.80–4.01)	<0.001 ***	1.57 (0.96–2.55)	0.071
Have you suffered from stigmatization because of your work?	No	Reference		Reference	
	Yes	2.88 (1.98–4.18)	<0.001 ***	1.37 (0.85–2.20)	0.193
Are you concerned about the increased number of COVID-19-positive patients or deaths?	No	Reference		Reference	
	Yes	1.88 (1.36–2.59)	<0.001 ***	1.12 (0.74–1.70)	0.584
Do you worry about the lack of personal protective equipment, which increases the risk of exposure when attending work?	No	Reference		Reference	
	Yes	2.19 (1.59–3.00)	<0.001 ***	1.33 (0.89–1.99)	0.163
Are you worried about the lack of a COVID-19 vaccine?	No	Reference		Reference	
	Yes	1.44 (1.06–1.94)	0.019 *	0.96 (0.67–1.39)	0.828
Are you worried about the lack of drugs to treat COVID-19?	No	Reference		Reference	
	Yes	1.81 (1.33–2.46)	<0.001 ***	1.24 (0.82–1.87)	0.303
Do you believe the understanding and support of your family members or peers have diminished due to the pandemic?	No	Reference		Reference	
	Yes	3.78 (2.26–6.33)	<0.001 ***	2.03 (1.11–3.68)	0.021 *
Drinking problem	No	Reference		Reference	
	Yes	1.68 (1.19–2.37)	<0.003 ***	1.39 (0.94–2.06)	0.096
Sleep quality	Good	Reference		Reference	
	Bad	3.29 (2.22–4.88)	<0.001 ***	2.44 (1.57–3.81)-	<0.001 ***

* *p* < 0.05; ** *p* < 0.01; *** *p* < 0.001.

**Table 5:** Stress odds ratio.

Variable		Univariable		Multivariable	
		OR (95% CI)	*p*-Value	OR (95% CI)	*p*-Value
Region	North, East and Islands	Reference		Reference	
	Central	0.80 (0.55–1.16)	0.243	1.00 (0.64–1.56)	0.999
	Southern	1.71 (1.05–2.79)	0.031 *	2.78 (1.50–5.14)	0.001 **
Age	20–30 years	Reference		Reference	
	31–40 years	1.73 (1.17–2.55)	0.006 **	1.25 (0.67–2.34)	0.486
	>40 years	1.66 (1.04–2.64)	0.033 **	1.13 (0.48–2.64)	0.785
Gender	Woman	Reference		Reference	
	Man	0.69 (0.34–1.37)	0.284	0.69 (0.30–1.59)	0.387
Marital status	Single	Reference		Reference	
	Divorce	0.88 (0.31–2.51)	0.814	0.49 (0.13–1.78)	0.278
	Married	1.34 (0.96–1.88)	0.084	0.94 (0.52–1.70)	0.837
Level of education	High school	Reference		Reference	
	College	1.32 (0.47–3.74)	0.597	0.93 (0.28–3.11)	0.911
	University	1.57 (0.56–4.44)	0.392	0.88 (0.26–2.97)	0.840
	Masters and Doctorate	1.43 (0.49–4.16)	0.512	0.61 (0.17–2.15)	0.438
History of disease	No	Reference		Reference	
	Yes	1.25 (0.42–3.79)	0.689	1.47 (0.38–5.67)	0.581
Any history of psychiatric illness in the past?	No	Reference		Reference	
	Yes	2.27 (0.56–9.16)	0.250	2.16 (0.43–10.76)	0.347
Work experience	≤5 years	Reference		Reference	
	6–10 years	1.68 (1.06–2.65)	0.028 *	1.29 (0.72–2.31)	0.395
	10–15 years	1.95 (1.25–3.05)	0.003 **	1.58 (0.77–3.27)	0.214
	≥15 years	1.58 (0.99–2.51)	0.054	1.11 (0.48–2.58)	0.813
Living with family members under the age of 18	No	Reference		Reference	
	Yes	1.53 (1.10–2.11)	0.011 *	0.95 (0.57–1.58)	0.841
Average working hours per week	≤72 h	Reference		Reference	
	72~96 h	1.67 (0.97–2.85)	0.063	1.61 (0.85–3.05)	0.141
	>96 h	2.09 (1.18–3.71)	0.012 *	1.92 (0.97–3.81)	0.061
Transport or interact with this many suspected or COVID-19-positive patients	≤20	Reference		Reference	
	21~40	1.82 (1.24–2.66)	0.002 **	1.60 (1.02–2.51)	0.040 *
	>40	1.94 (1.17–3.21)	0.01 *	1.20 (0.97–3.81)	0.571
Do you worry about spreading the disease to your family due to work during the COVID-19 pandemic?	No	Reference		Reference	
	Yes	3.02 (1.60–5.69)	0.001 **	1.89 (0.87–4.11)	0.110
Do you want to find alternate accommodation and temporarily live apart from your family because of your work during the COVID-19 pandemic?	No	Reference		Reference	
	Yes	2.21 (1.59–3.07)	<0.001 ***	1.38 (0.93–2.06)	0.111
Have you experienced violence because of work?	No	Reference		Reference	
	Yes	2.78 (1.88–4.13)	<0.001 ***	1.49 (0.89–2.47)	0.127
Have you suffered from stigmatization because of your work?	No	Reference		Reference	
	Yes	3.24 (2.24–4.70)	<0.001 ***	1.40 (0.85–2.29)	0.186
Are you concerned about the increased number of COVID-19-positive patients or deaths?	No	Reference		Reference	
	Yes	2.35 (1.63–3.39)	<0.001 ***	1.56 (0.97–2.52)	0.065
Do you worry about the lack of personal protective equipment, which increases the risk of exposure when attending work?	No	Reference		Reference	
	Yes	2.07 (1.45–2.93)	<0.001 ***	1.06 (0.68–1.67)	0.795
Are you worried about the lack of a COVID-19 vaccine?	No	Reference		Reference	
	Yes	1.45 (1.04–2.00)	0.026 *	1.02 (0.68–1.53)	0.930
Are you worried about the lack of drugs to treat COVID-19?	No				
	Yes	1.77 (1.26–2.48)	0.001 **	0.98 (0.62–1.56)	0.947
Do you believe the understanding and support of your family members or peers have diminished due to the pandemic?	No	Reference		Reference	
	Yes	6.06 (3.68–10.00)	<0.001 ***	3.27 (1.83–5.86)	<0.001 ***
Drinking problem	No	Reference		Reference	
	Yes	1.44 (1.00–2.06)	0.049 *	1.04 (0.68–1.60)	0.843
Sleep quality	Good	Reference		Reference	
	Bad	5.02 (3.39–7.42)	<0.001 ***	4.34 (2.76–6.82)	<0.001 ***

* *p* < 0.05; ** *p* < 0.01; *** *p* < 0.001.

## Data Availability

Please contact the corresponding author.

## Supplementary Materials

The following supporting information can be downloaded at: https://www.mdpi.com/article/10.3390/ijerph20010137/s1. Table S1: The proportion of people with moderate or higher levels of depression, anxiety and stress.
