# Supplementary material for: Investigation of Psychological Stress and Sleep Quality of Emergency Medical Technicians in Taiwan Fire Department during the COVID-19 Pandemic
Source: Int J Environ Res Public Health. 2022 Dec 22;20(1):137. doi: 10.3390/ijerph20010137 (PMC9819878; doi:10.3390/ijerph20010137)
Supplement: Supplementary file 1 [file ijerph-20-00137-s001.zip › ijerph-2075525-SI.pdf]

Supplementary Table S1. The proportion of people with moderate or higher levels of depression, anxiety and stress.

Variable N(%)		Depression		Anxiety		Stress	
		<14	≥14	<10	≥10	<19	≥19
Total		404(58.7%)	284(41.3%)	377(54.8%)	311(45.2%)	476(69.2%)	212(30.8%)
Region	North, East, and Islands	238(61.0%)	152(39.0%)	220(56.4%)	170(43.6%)	270(69.2%)	120(30.8%)
	Central	130(59.9%)	87(40.1%)	124(57.1%)	93(42.9%)	160(73.7%)	57(26.3%)
	Southern	36(44.4%)	45(55.6%)	33(40.7%)	48(59.3%)	46(56.8%)	35(43.2%)
Age	20- 30 years	156 (72.9%)	58 (27.1%)	145(67.8%)	69(32.2%)	164(76.6%)	50(23.4%)
	31- 40 years	174 (53.5%)	151(53.2%)	162(49.8%)	163(50.2%)	213(65.5%)	12(34.5%)
	>40 years	74(49.7%)	75(50.3%)	70(47.0%)	79(53.0%)	99(66.4%)	50(33.6%)
Gender	Woman	17(47.2%)	19(52.8%)	11(30.6%)	25(69.4%)	22(61.1%)	14(38.9%)
	Man	387(59.4%)	265(40.6%)	366(56.1%)	286(43.9%)	454(69.6%)	198(30.4%)
Marital status	Single	183(63.5%)	105(36.5%)	174(60.4%)	114(39.6%)	209(72.6%)	79(27.4%)
	Divorce	12(60.0%)	8(40.0%)	9(45.0%)	11(55.0%)	15(75.0%)	5(25.0%)
	Married	209(55.0%)	171(45.0%)	194(51.1%)	186(48.9%)	252(66.3%)	28(33.7%)
Level of education	High school	14(66.7%)	7(33.3%)	14(66.7%)	7(33.3%)	16(76.2%)	5(23.8%)
	College	171(63.3%)	99(36.7%)	147(54.4%)	123(45.6%)	191(70.7%)	79(29.3%)
	University	142(54.4%)	119(45.6%)	137(52.5%)	124(47.5%)	175(67.0%)	86(33.0%)
	Masters and Doctorate	77(56.6%)	59(43.4%)	79(58.1%)	57(41.9%)	94(69.7%)	42(30.9%)
History of disease	No	397(58.9%)	277(41.1%)	369(54.7%)	305(45.3%)	467(69.3%)	207(30.7%)
	Yes	7(50.0%)	7(50.0%)	8(57.1%)	6(42.9%)	9(64.3%)	5(35.7%)
Any history of psychiatric illness in the past?	No	400(58.8%)	280(41.2%)	374(55.0%)	306(45.0%)	472(69.4%)	208(30.6%)
	Yes	4(50.0%)	4(50.0%)	3(37.5%)	5(62.5%)	4(50.0%)	4(50.0%)
Work experience	≤5 years	153(72.2%)	59(27.8%)	142(67.0%)	70(33.0%)	162(76.4%)	50(23.6%)
	6- 10 years	86(55.5%)	69(44.5%)	81(52.3%)	74(47.7%)	103(66.5%)	52(33.5%)
	11-15 years	78(47.3%)	87(52.7%)	73(44.2%)	92(55.8%)	104(63.0%)	61(37.0%)

Variable N(%)		Depression		Anxiety		Stress	
		**<14**	**≥14**	**<10**	**≥10**	**<19**	**≥19**
Total		404(58.7%)	284(41.3%)	377(54.8%)	311(45.2%)	476(69.2%)	212(30.8%)
	>15 years	83(54.6%)	69(45.4%)	77(50.7%)	75(49.3%)	103(67.6%)	49(32.2%)
Living with family members under the age of 18	No	244(63.5%)	140(36.5%)	232(60.4%)	152(39.6%)	281(73.2%)	103(26.8%)
	Yes	160(52.6%)	144(47.4%)	145(47.7%)	159(52.3%)	195(64.1%)	109(35.9%)
Living with family members over the age of 65	No	307(61.2%)	195(38.8%)	285(56.8%)	217(43.2%)	357(71.1%)	145(28.9%)
	Yes	97(52.2%)	89(47.8%)	92(49.5%)	94(50.5%)	119(64.0%)	67(36.0%)
Average working hours per week	≤72 hours	66(69.5%)	29(30.5%)	61(64.2%)	34(35.8%)	75(78.9%)	20(21.1%)
	72~96 hours	229(57.3%)	171(42.8%)	217(54.3%)	183(45.8%)	277(69.3%)	123(30.8%)
	>96 hours	109(56.5%)	84(43.5%)	99(51.3%)	94(48.7%)	124(64.2%)	69(35.8%)
The work unit is dedicated to transporting confirmed or suspected infected patients	No	298(58.5%)	211(41.5%)	278(54.6%)	231(45.4%)	353(69.4%)	156(30.6%)
	Yes	106(59.2%)	73(40.8%)	99(55.3%)	80(44.7%)	123(68.7%)	56(31.3.%)
Transport or interact with this many suspected or COVID-19-positive patients	≤20	285(62.8%)	169(37.2%)	266(58.6%)	188(41.4%)	335(73.8%)	119(26.2%)
	21~40	85(53.8%)	73(46.2%)	79(50.0%)	79(50.0%)	96(60.8%)	62(39.2%)
	>40	34(44.7%)	42(55.3%)	32(42.1%)	44(57.9%)	45(59.2%)	31(40.8
Did you contract the severe acute respiratory syndrome (SARS) in 2002?	No	337(59.0%)	234(41.0%)	318(55.7%)	253(44.3%)	395(69.2%)	176(30.8%)
	Yes	67(57.3%)	50(42.7%)	59(50.4%)	58(49.6%)	81(69.2%)	36(30.8%)
Do you worry about spreading the disease to your family due to work during the COVID-19 epidemic?	No	59(69.4%)	26(30.6%)	62(72.9%)	23(27.1%)	73(85.9%)	12(14.1%)
	Yes	345(57.2%)	258(42.8%)	315(52.2%)	288(47.8%)	403(66.8%)	200(33.2%)
Do you want to find alternate accommodation and temporarily live apart from your family because of your work during the COVID-19 pandemic?	No	239(61.6%)	149(38.4%)	233(60.1%)	155(39.9%)	297(76.5%)	91(23.5%)
	Yes	165(55.0%)	135(45.0%)	144(48.0%)	156(52.0%)	179(59.7%)	121(40.3%)

Variable N(%)		Depression		Anxiety		Stress	
		<14	≥14	<10	≥10	<19	≥19
Total		404(58.7%)	284(41.3%)	377(54.8%)	311(45.2%)	476(69.2%)	212(30.8%)
Have you experienced violence because of work?	No	350(62.5%)	210(37.5%)	332(59.3%)	228(40.7%)	412(73.6%)	148(26.4%)
	Yes	54(42.2%)	74(57.8%)	45(35.2%)	83(64.8%)	64(50.0%)	64(50.0%)
Have you suffered from stigmatization because of your work?	No	338(63.4%)	195(36.6%)	323(60.6%)	210(39.4%)	401(75.2%)	132(24.8%)
	Yes	66(42.6%)	89(57.4%)	54(34.8%)	101(65.2%)	75(48.4%)	80(51.6%)
Are you worried about the frequent reports of COVID-19 in the media?	No	199(58.2%)	143(41.8%)	196(57.3%)	146(42.7%)	247(72.2%)	95(27.8%)
	Yes	205(59..2%)	141(40.8)	181(52.3%)	165(47.7%)	229(66.2%)	117(33.8%)
Are you concerned about the increased number of COVID-19-positive patients or deaths?	No	152(61.8%)	94(38.2%)	159(64.6%)	87(35.4%)	197(80.1%)	49(19.9%)
	Yes	252(57.0%)	190(43.0%)	218(49.3%)	224(50.7%)	279(63.1%)	163(36.9%)
Do you worry about the lack of personal protective equipment, which increases the risk of exposure when attending work?	No	180(66.7%)	90(33.3%)	179(66.3%)	91(33.7%)	211(78.1%)	59(21.9%)
	Yes	224(53.6%)	194(46.4%)	198(47.4%)	22(52.6%)	265(63.4%)	153(36.6%)
Are you worried about the lack of a COVID-19 vaccine?	No	229(60.1%)	152(39.9%)	224(58.8%)	157(41.2%)	277(72.7%)	104(27.3%)
	Yes	175(57.0%)	132(43.0%)	153(49.8%)	154(50.2%)	199(64.6%)	108(35.2%)
Are you worried about the lack of drugs to treat COVID-19?	No	182(63.2%)	106(36.8%)	182(63.2%)	106(36.8%)	219(76.0%)	69(24.0%)
	Yes	222(55.5%)	178(44.5%)	195(48.8%)	205(51.2%)	257(64.3%)	143(35.8%)
Do you believe the understanding and support of your family members or peers have diminished due to the epidemic?	No	381(62.8%)	226(37.2%)	355(58.5%)	252(41.5%)	450(74.1%)	157(25.9%)
	Yes	23(28.4%)	58(71.6%)	22(27.2%)	59(72.8%)	26(32.1%)	55(67.9%)
Drinking problem	No	312(61.1%)	199(38.9%)	297(58.1%)	214(41.9%)	364(71.2%)	147(28.8%)
	Yes	92(52.0%)	85(48.0%)	80(45.2%)	97(54.8%)	112(63.3%)	65(36.7%)
Sleep quality	Good	367(67.1%)	180(32.9%)	332(60.7%)	215(39.3%)	420(76.8%)	127(23.2%)
	Poor	37(26.2%)	1.4(73.8%)	45(31.9%)	96(68.1%)	56(39.7%)	85(60.3%)
